# Hospital-Based Air-Borne and Surface-Borne Bacterial Pathogens and Their Antimicrobial Profiles in Wolaita Sodo, Southern Ethiopia

**DOI:** 10.1155/2022/5718341

**Published:** 2022-10-31

**Authors:** Chemere Madebo, Admasu Haile, Temesgen Eticha, Fithamlak Solomon

**Affiliations:** ^1^Areka Urban Development and Construction, Wolaita, Ethiopia; ^2^Department of Medical Laboratory Sciences, College of Medicine and Health Science, Wolkite University, Wolkite, Ethiopia; ^3^School of Medical Laboratory Sciences, College of Medicine and Health Sciences, Wolaita Sodo University, Wolaita Sodo, Ethiopia

## Abstract

**Background:**

It is well documented that hospital environments are the niche/reservoir of many clinically important microorganisms, including multidrug-resistant air-borne and surface-borne pathogens. This problem is the most pressing public health concern, particularly in developing countries like Ethiopia, due to its poor infection management system. This study was planned to detect air-borne and surface-borne bacterial pathogens and their antimicrobial resistance patterns in Wolaita Sodo University Comprehensive Hospital, Southern Ethiopia.

**Method:**

A laboratory-based cross-sectional study was conducted from May to July 2021. Swabbing and open-plate sample collection methods were used to collect specimens. Standard bacteriological techniques were used to isolate and identify bacterial pathogens. The Mueller-Hinton agar was used to detect the drug susceptibility pattern of bacteria by using the Kirby-Bauer disc diffusion method.

**Result:**

From a total of 323 samples tested, 118 (36.5%) showed the growth of bacteria. The detection rate of bacterial pathogens in the intensive care unit (35.4%) was higher than in operation theater. From the total of 118 bacterial isolates, 39.8%, 27.9%, 20.3%, and 11.5% of *S. aureus*, *P. aeruginosa*, *Klebsiella pneumoniae*, and *E. coli*, respectively, were surface-borne. Whereas 37%, 25%, 20.83, and 16.6% of *S. aureus*, *P. aeruginosa*, *Klebsiella species*, and *E. coli*, respectively, were air-borne. *S. aureus* showed a 19.04 to 80.9% range of antimicrobial resistance to different classes of antibiotics from surface specimens. A 12.5–100% range of antibiotic resistance levels was detected for all Gram-negative surface-borne bacterial pathogens. *P. aeruginosa* was 66.7%, 73.3%, and 73.3% resistant to gentamicin, chloramphenicol, and ceftriaxone, respectively. *K. pneumoniae* showed 75% and 87.5% resistance to ceftriaxone and ciprofloxacin, respectively, and a completely ampicillin-resistant *E. coli* was detected. From a total of 48 bacterial pathogens identified from surfaces in the intensive care unit, 34 (70.8%) developed multidrug resistance.

**Conclusion:**

A significant prevalence of surface-borne bacterial pathogens was detected. This study revealed that *S. aureus*, *P. aeruginosa*, *K. pneumoniae*, and *E. coli* were nosocomial infection concerns of the hospital, and this could be the reason for different types of hospital acquired infections in the study area. A high prevalence of MDR was detected in the most surface-borne bacterial isolates.

## 1. Introduction

Infectious diseases are still a major public health problem in hospital environments and in the population generally. The high prevalence of bacterial organisms like methicillin-resistant *Staphylococcus aureus* (MRSA), *Clostridium difficile*, TB infections, and other bacteria with antibiotic resistance requires increasing effort for containment of organisms [1–4].

Nosocomial infections affect approximately 5–10% of patients in developed countries, but the risk is 2 to 20 times higher in developing countries and their presence induces an extension in the length of hospitalizations. Overuse of antibiotics leads sometimes to the emergence of resistant microorganisms [5]. The main reservoirs of infections include patients' environment, such as hospital rooms' surfaces and medical or accommodation equipment [5].

Generally, most hospital-acquired infections are transmitted from person to person (healthcare workers to patients) directly or through intermediate objects, which are often inanimate. Endogenous transmission of patients' own organisms from one part of the body to another may result in nosocomial infection in patients in ICU and OT [6–8].

The maintenance of air quality in hospitals and medical centers has become a critical part of hospital management protocols to prevent air pollutants [9, 10]. Different studies indicate that bacterial transmission between patients, staff, and the inanimate environment is significant. The environment may have more effect on intensive care units (ICUs) and operation theater (OT) because of patients' unstable clinical status that predisposes them to infections [11, 12]. The varying prevalence and antimicrobial profiles of bacterial isolates from the air and surfaces of hospitals are indicated by many studies in Ethiopia [13–17].

Antimicrobial resistance is a global threat with a high burden in developing countries, where the infectious disease burden is high and a low economy prevents the widespread application of newer, more expensive agents. Nosocomial infections are one of the leading causes of disease and death in the developing world, and management of them has been critically compromised by the appearance and rapid spread of resistant strains of pathogens [18–20]. Antimicrobial resistance results in reduced efficacy of antimicrobial agents and leads to poor treatment outcomes. Detection of the magnitude of the antimicrobial problem and its impact on human health, including costs for healthcare due to AMR, still largely needs further systematic investigation [21]. Updated knowledge of local and regional antimicrobial resistance (AMR) is critically important for the maintenance of quality air and management protocols in hospital environments. Different studies have been conducted to identify antimicrobial profiles from bacterial isolates from hospitals in clinical specimens [22–30], but little is known about the prevalence and antimicrobial profiles of bacterial pathogens in inanimate objects, so this study was aimed at determining bacterial pathogens and antimicrobial profiles in Wolaita Sodo town, Southern Ethiopia.

## 2. Materials and Methods

### 2.1. Study Setting

**Figure 1:**
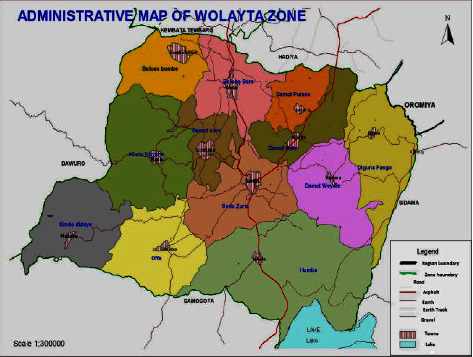
Map of study setting (Source: https://touristinfo.ning.com/photo/damot-mountain-27.

The study was conducted at Wolaita Sodo University Comprehensive Hospital (WSUCH), Wolaita Sodo, Ethiopia. Wolaita Sodo, the administrative town of Wolaita zone, is located about 330 km away from Addis Ababa, the capital of Ethiopia, as shown in Figure 1. According to the CSA projection in 2012, the total population the hospital serves is above 2 million people in the catchment area. It has 370 beds for inpatient service, which includes surgical, gynecology, medical, obstetrics, intensive care unit, operation theater, outpatient department, and DRW. The hospital is purposefully selected based on its general hygiene profile and the presence of conducive habitats for the growth of air-borne and surface bacterial pathogens of medical importance.

### 2.2. Study Design and Period

A Hospital-based cross-sectional study was conducted at the intensive care unit and operation theater room of WSUCH, from May to July 2021.

### 2.3. Sample Size Determination and Sampling Techniques

The sample size was calculated using a single population proportion formula considering the 95% level taking the prevalence of 70% from a previous study [31] and degree of allowable error 0.05. The sample size is therefore calculated as follows:

(1) $$\begin{array}{c} \text{Sample size}\left(n\right)=\frac{z^{2}\left(P\right)\left(1-P\right)}{d^{2}}\text{,} \end{array}$$

where *N* = sample size. *Z* = statistics for level of confidence = 1.96. *D* = desired level of precision, 0.05 and *P* = estimated prevalence = 0.7. *N* = (1.96)^2^ × 0.7(1–0.7) = 323.

Estimated sample size was 323, collected consecutively from ICU and OT wards until the required samples size met.

### 2.4. Specimen Collection

The specimen was collected from the air and surfaces of the floor, patients' beds, walls, door handles, chairs, ward sinks, window handles, light switches, bedsheets, and catheters. The air samples were collected in the daytime (at 8-9 AM, 11 AM-12 PM, and 4-5 PM) considering the fact that a higher patient, staff, and attendee load could result in a higher chance for acquiring infection through the airway. It was collected using settle plate sampling method following 1/1/1 schedules [(a 9 cm in diameter sterile Petri dish with 5% blood agar [(9 mm diameter)] was left open to the air for an hour, a meter above the floor and a meter from the wall [32]. Indoor air samples were collected from the intensive care unit (ICU) and operation theater (OT). During air sampling, protective gowns, sterile gloves, and mouth masks were worn to prevent self-contamination of the 5% blood agar plate. Then Petri dish plates were labeled with the sample number, selected ward, date, and time of sample collection. Four agar plates were placed in each of the selected wards, 4 m apart. Almost immediately after the collection, samples were transported to the microbiology laboratory in sealed plastic bags and incubated aerobically for 24 hours at 37c0. Swabbing was employed to collect samples from floors, walls, patients' beds, door handles, light switches, chairs, catheters, ward sinks, bed sheets, and window handles of the hospital, and swabs were placed into test tubes containing 0.5 ml of normal saline solution and transported to the microbiology laboratory within 30 minutes to 1 hour of collection [33].

Preliminary identification was done by taking colonies from Mannitol salt agar, MacConkey agar, and inoculating them onto blood agar plates 5% (BAP), nutrient agar, and Mueller-Hinton agar. The inoculated agar plates were incubated at 37°C for 24 hours. Pigment production was confirmed by sub-culturing suspected colonies on nutrient agar and Mueller-Hinton agar (oxide. England) WHO, 2010. Further identification of *S. aureus, E. coli, P. aeruginosa, and Klebsiella species* was done using colony morphology, Gram stain, and conventional biochemical tests such as triple sugar iron (TSI) tests, SIM tests, catalase tests, coagulase tests, citrate tests, and oxidase tests.

### 2.5. Transport of Specimens and Specimens Processing

Surface samples of walls, door handles, ward sinks, patient beds, catheters, chairs, bed sheets, window handles, floors, and light switches of ICU and OT rooms were collected by swabbing and placing them into test tubes containing 0.5 ml of saline solution. It is then transported to the central microbiology laboratory within 30 minutes to 1 hour of collection and incubated at 37°C for 24 hours.

### 2.6. Microbiological Activities

Blood agar, MacConkey agar, Mannitol salt agar, nutrient agar, and Mueller-Hinton agars were culture media used for bacterial cultivation. After the Gram staining method was done, different biochemical tests like catalase, coagulase, oxidase, Simmon's citrate, triple sugar agar, and sulfide indole motility were used to identify bacterial species.

### 2.7. Selection of Antimicrobials and Antimicrobial Susceptibility Test

Antibiotics were selected based on local availability, literature information, and effectiveness. The grades of susceptibility pattern were recognized as resistant, intermediate, and sensitive by comparison of the zone of inhibition as indicated in CLSI 2020. An antimicrobial susceptibility test was done for gentamicin (10 *µ*g), amikacin (30 *µ*g), ciprofloxacin (5 *µ*g), cefepime (30 *µ*g), norfloxacin (10 *µ*g), doxycycline (30 *µ*g), tetracycline (30 *µ*g), vancomycin (30 *µ*g), cloxacillin (5 *µ*g), clindamycin (10 *µ*g), metronidazole (5 *µ*g), tobramycin (10 *µ*g), kanamycin (30 *µ*g), and penicillin (10 *µ*g) by the disc diffusion method.

### 2.8. Data Analysis

The processing of data was done by computer software. The data were coded and entered into an MS Excel spreadsheet and checked for accuracy. After validation, it was transferred and processed using computer software SPSS version 21 for analysis. Pearson's chi-square tests were used when appropriate to analyze the proportion of categorical data. The 95% confidence level was used, and results were considered statistically significant at (*p* < 0.05).

### 2.9. Ethical Considerations

The study was approved by the ethical review committee of Wolaita Sodo University and permission was obtained to conduct the research on inanimate objects in hospital wards. The results of the study were communicated to the responsible bodies for any beneficiary measures.

## 3. Results

### 3.1. Distribution of Bacterial Pathogens

**Figure 2:**
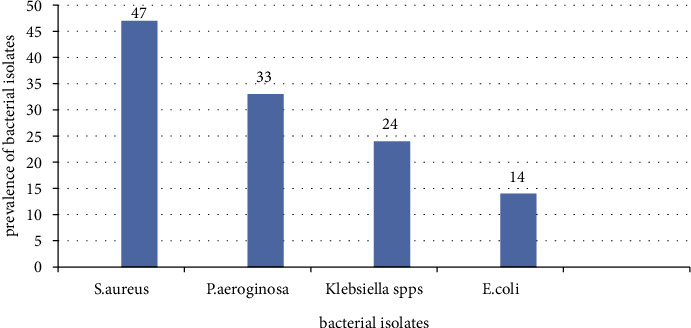
Distribution of air-borne and surface-borne bacterial isolates at WSUCH, Wolaita Sodo, Ethiopia, 2021.

A total of 5 bacterial isolates were detected in this study from a total of 323 specimens collected, from which 227 surface and 96 air (settle plate) samples were collected from the intensive care unit (ICU) and the operation theater (OT). Of the total different specimens collected, 118(36.53%) samples showed bacterial growth on culture media, categorized into 4 genera/species: *Staphylococcus, Pseudomonas, Escherichia, and Klebsiella pneumoniae*. The predominant bacterium was *S. aureus* (47) (39.8%), followed by *P. aeruginosa* (33) (27.96%), as shown in Figure 2.

#### 3.1.1. Prevalence of Bacteria in Surfaces over ICU Ward

From total positive cases, a large proportion (48) (40.67%) of bacteria was detected from surface samples of the ICU, with a relatively high prevalence from patient bed surfaces (8) (16.67%) and door handles (7) (14.5%). The low bacterial pathogen was detected from surfaces of catheters (1) (4.1%) and light switches (2.08%). *S. aureus* and *P. aeruginosa* were the predominant pathogens isolated from surface samples of the ICU with a prevalence of 21 (43.75%) and 15 (31.25%), respectively, as shown in Table 1.

#### 3.1.2. Distribution of Bacterial Pathogens in Surfaces over Operation Theater Ward

The total bacterial isolate from surface samples of OT was comparable proportion with bacteria isolated from surface samples of ICU (46 vs. 48). The predominant bacteria identified were *S. aureus* 17 (36.95), followed by *P. aeruginosa* 12 (26.08%) from the surfaces of the OT room. A large proportion (8) (17.4%) of bacteria was isolated from the walls of the OT ward, followed by the patient bed surface (15.2%) (7/46) and door handles (15.2%) (7/46). No bacteria were detected from the surface of the light switches of the OT room (Table 2).

#### 3.1.3. Prevalence of Bacterial Pathogen in Air of ICU and OT

**Figure 3:**
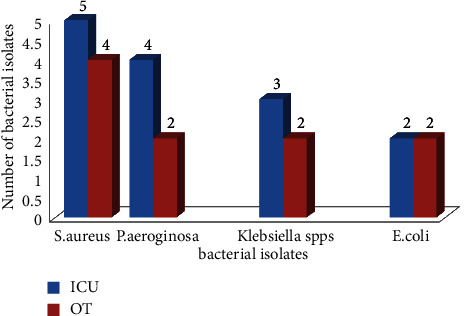
Distribution of air-borne bacterial isolates from ICU and OT wards at WSUCH, Wolaita Sodo, Ethiopia, 2021.

From a total of 118 bacterial isolates detected, 24 (25%) bacterial growth was detected in air specimens in ICU and OT wards, with a relatively high prevalence in ICU of 58.34% (14/24). Of 24 isolates from air specimens, *S. aureus* accounted for 9 (37.5%), followed by *P. aeruginosa* at 6 (25%). Bacterial isolates were detected mostly from ICU wards with the exception of *E. coli*, which was equal in both ICU and OT air samples as shown in Figure 3.

#### 3.1.4. Antimicrobials Profiles of Bacterial Isolates Detected from Air Sample

As shown in Table 3, a varying antimicrobial resistance pattern of bacterial isolates was detected with regard to sample sources (air vs surface). A low resistance pattern of *S. aureus* was recorded for ciprofloxacin (22.2%), amikacin (22.2%), ampicillin (22.2%), ceftriaxone (22.2%), and cefepime (33.3%). *S. aureus* showed high resistance to chloramphenicol and penicillin, 88.8% to both antimicrobials. 83.3% and 66.7% susceptibility to ciprofloxacin and amikacin, respectively, was detected for *P. aeruginosa*, but it showed complete resistance to penicillin and 83.3%, 66.7%, and 66.7% to cefepime, ampicillin, and ceftriaxone, respectively. The antimicrobial resistance pattern of *E. coli* was 75% to doxycycline and ceftriaxone, where it showed 75% sensitivity to ciprofloxacin.

#### 3.1.5. Antimicrobial Profiles of Bacterial Isolated from Surface Samples

The antimicrobial susceptibility proportion of *S. aureus* is 94.6% (35/38) to ampicillin, 68.4% (26/38) to chloramphenicol, and 60.5% (23/38) to ciprofloxacin. *S. aureus* showed higher resistance to penicillin at 89.2% (33/38) and gentamicin at 62.2% (23/38). The antimicrobial resistance of *P. aeruginosa* species was 89.2% and 64.9% susceptible to ciprofloxacin and ampicillin, respectively. This study showed that *P. aeruginosa* developed high resistance to chloramphenicol, 82.1%, followed by cefepime (71.4%), and gentamicin (60.7%). *Klebsiella species* showed 63.1%, 68.4%, 68.4%, and 84.2% sensitivity to amikacin, gentamicin, cefepime, and ciprofloxacin, respectively, and 26.3% resistance to two antimicrobials (gentamicin, amikacin). Ciprofloxacin and cefepime were drugs effective against *E. coli* that showed 70% and 80% susceptibility, whereas amikacin and doxycycline showed 70% and 80% resistance, respectively, as presented in Table 4.

### 3.2. Multi-Drug Resistance Pattern of Surface-Borne Bacterial Isolates

**Figure 4:**
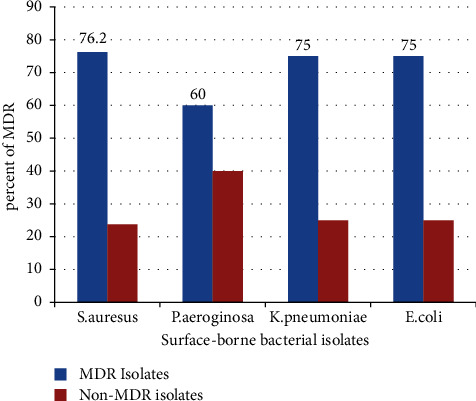
Surface-borne multidrug resistant bacterial isolates at WSUCH, Wolaita Sodo, Ethiopia, 2021.

The overall prevalence of multidrug resistance (MDR) was 70.8%, of which 47.05% was Gram-positive and 52.95 were Gram-negative. 60%, 75%, 75%, and 76.2% of *P. aeruginosa*, *K. pneumonia, E. coli*, and *S. aureus*, respectively, developed MDR from total Figure 4.

## 4. Discussion

This study was aimed at detecting bacterial isolates and their antimicrobial profiles in air and surface specimens in Wolaita Sodo University teaching and referral hospital. The overall prevalence of bacterial growth in this study was 36.53%. This finding was comparable with studies done in Northwest Ethiopia (39.6%) and Uganda (44.2%) [34]. However, this finding was higher than previous studies done in Morocco (26.8%) [35], Palestine (24.7%) [36], and Egypt (25.6%) [37]. In contrast, this finding was lower than previous studies conducted in Ethiopia (70%) [31], Northern Ethiopia [15], Jimma (66%) [16], Eastern Ethiopia (53.8%), Addis Ababa (86%) [38], Zimbabwe (86.21%) [39], and Nigeria (56.7%) [40]. This variation could be due to differences in the infection management systems of hospitals within or in different countries, hygiene practices of the population in the hospital environment, architecture characteristics, sample size, and microbial characteristics.

In this study, 4 bacterial species, *S. aureus*, *P. aeruginosa*, *K. pneumoniae,* and *E. coli*, with 39.8%, 27.96%, 20.34%, and 11.86%, respectively, from both air and surface specimens, indicated that *S. aureus* was the predominant isolate in this study. This finding was supported by another study conducted in eastern Ethiopia which reported a 35% prevalence of *S. aureus* [41], and Uganda in which *S*. *aureus* was the leading contaminant of the hospital [34]. In contrast to this finding, another study in Ethiopia reported a high prevalence of CoNS (42.9%) and *S. aureus* (20.3%) [42], but the study agreed with our study as it detected a low prevalence of *E. coli.* The increased prevalence of *S. aureus* might be due to the bacterial resistance ability to dry conditions of the hospital environment and transmission from skin, nasal, and boils of healthcare workers and patients.

The current study with regard to specimen type detected bacterial isolates mostly from surfaces of hospitals with a 79.67% greater than air (20.33%). However, this finding was contradicted by another study done in Ethiopia [17] that reported relatively higher bacterial isolates from the air than surfaces (53.7% vs. 46.3%). Bacterial contamination in the ICU room was comparable with that in the OT room in this study (48.9% vs. 51.1%). The surfaces over the ICU yielded varying bacterial growth. In this regard, bed linens, door handles, ward sinks, and walls have 16.6% (8/48), 14.5% (7/48), 12.5% (6/48), and 14.5% (6/48), respectively. In the OT ward, the surface of light switches showed no bacterial growth, and window handles and catheters showed low 4.3% (2/46) bacterial growth.

In developing countries including Ethiopia, the majority of health professionals lack up-to-date knowledge on the pattern of antimicrobials due to the absence of local antibiogram data [43, 44]; so in this study, we performed antimicrobial resistance and sensitivity pattern of isolated bacteria. *S. aureus* showed a high 88.8% resistance to penicillin and chloramphenicol in the present study. Similar findings were found in Ethiopia with 82.7% and 87% penicillin resistance to *S. aureus* [8, 45].*S. aureus* was 62.2% sensitive to ciprofloxacin, 67.6% to ceftriaxone, and 70.3% to chloramphenicol. This was supported by a study done in Addis Ababa, with 78.6% and 64.3% sensitivity to chloramphenicol and ciprofloxacin, respectively [46]. However, resistance is lower than in a study done in Arbamich that showed complete resistance to penicillin [47]. Ciprofloxacin, ampicillin, ceftriaxone, and cefepime were antimicrobial agents that showed low resistance (22.2%) to *S. aureus.*

Effective antibiotics against *P. aeruginosa* in the current study were ciprofloxacin and amikacin, with 83.3% and 66.7%, respectively. This result was compared with another study in Ethiopia that reported 85.7% susceptibility to ciprofloxacin. However, the same study found, contrarily, 57.1% resistance to amikacin [46]. *P. aeruginosa* showed complete resistance to penicillin and 83.3%, 66.7%, and 66.7% resistant to cefepime, ampicillin, and ceftriaxone, respectively. *P. aeruginosa* from surfaces showed 89.2%, 64.9%, and 53.6% sensitivity to ciprofloxacin, ampicillin, and ceftriaxone, respectively, and 71.4% and 82.1% resistance to cefepime and chloramphenicol in this study. *The Klebsiella species* showed 80% sensitivity to ciprofloxacin and ceftriaxone and 60% to amikacin and gentamicin. Lower sensitivity (40%) to ciprofloxacin and complete susceptibility (100%) to amikacin was found in a study done in Arbamich, Ethiopia [47]. *E. coli* isolated from the air was 75% resistant to doxycycline and ceftriaxone but 75% susceptible to ciprofloxacin. Whereas relatively effective antibiotics to surface isolated *E. coli* ciprofloxacin, cefepime, and ampicillin with ≥70% sensitivity, it showed 70% resistance to amikacin and 80% to doxycycline. An intermediate antimicrobial resistance pattern was detected for all bacterial isolates in this study, and this agreed with other studies done in Ethiopia [47]. The variation in antimicrobial susceptibility and resistance among bacteria might be due to different mechanisms of resistance in general and the study setting in particular.

## 5. Conclusion

A significant prevalence of surface-borne bacterial pathogens was detected. This study revealed that *S. aureus*, *P. aeruginosa*, *K. pneumoniae*, and *E. coli* were nosocomial infection concerns of the hospital, and this could be the reason for different types of hospital-acquired infections in the study area. The overall prevalence of multidrug resistance (MDR) was 70.8%, of which 47.05% was Gram-positive and 52.95 were Gram-negative.

## Figures and Tables

**Table 1:** Frequency of bacterial isolates from surface of ICU at WSUCH, Wolaita Sodo Ethiopia, 2021.

Source of specimens	Type of bacterial isolated				
	*S. aureus n = 21*	*P. aeruginosa n = 15*	*E. coli n = 4*	*Klebsiella spp*. *n = 8*	Total *n* = 48
Door handles	3 (14.2%)	3 (20%)	—	1 (12.5%)	7 (14.5%)
Walls	3 (14.2%)	2 (13.3%	1 (25%)	—	6 (12.5%)
Floor	2 (9.5%)	2 (13.3%)	—	1 (12.5%)	5 (10.4%)
Window handles	1 (4.7%)	1 (6.6%)	—	1 (12.5%)	3 (6.25%)
Patient bed surfaces	4 (19.04%)	1 (6.6%)	1 (25%)	2 (25%)	8 (16.6%)
Catheters	—	1 (6.6%)	1 (25%)	—	2 (4.1%)
Light switches	1 (4.75%)	—	—	—	1 (2.08%)
Chair	2 (9.5%)	1 (6.6%)	1 (25%)	1 (12.5%)	5 (10.4%)
Ward sinks	2 (9.5%)	3 (20%)	—	1 (12.5%)	6 (12.5%)
Bed sheets	3 (14.2%)	1 (6.6%)	—	1 (12.5%)	5 (10.4%)

**Table 2:** Distribution of bacterial isolates in surfaces over operation theater ward, WSUCH, Wolaita Sodo, Southern Ethiopia, 2021.

Source of specimens	Type of bacterial isolated				
	*S. aureus n = 17*	*P. aeruginosa n = 12*	*E. coli n = 6*	*K. pneumoniae n = 11*	Total *n* = 46
Door handles	3 (17.6%)	2 (16.6%)	1 (16.6%)	1 (9.09%)	7 (15.2%)
Floors	2 (11.7%)	1 (8.3%)	1 (16.6%)	2 (18.1%)	6 (13.04%)
Window handles	1 ( (5.8%)	—	—	1 (9.09%)	2 (4.3%)
Patient bed surfaces	3 (17.6%)	2 (16.6%)	1 (16.6%)	1 (9.09%)	7 (15.2%)
Chair	2 (11.7%)	1 (8.3%)	—	1 (9.09%)	4 (8.69%)
Bed sheets	2 (11; 7%)	2 (16.6%)	—	1 (9.09%)	5 (10.86%)
Light switches	—	—	—	—	—
Walls	3 (17.6%)	2 (16.6%)	1 (16.6%)	2 (18.1%)	8 (17.39%)
Catheters	—	—	1 (16.6%)	1 (9.09%)	2 (4.3%)
Ward sinks	1 (5.8%)	2 (16.6%)	1 (16.6%)	1 (9.09%)	5 (10.86%)
Total	17 (100%)	12 (100%)	6 (100%))	11 (100%)	46 (100%)

**Table 3:** Antimicrobial resistance pattern of air-borne bacterial isolates at WSUCH, Wolaita Sodo, Ethiopia, 2021.

Bacterial Isolates	Pattern	Antimicrobial agents								
		GEN	CIP	AMA	DOX	CEP	CAF	PEN	AMP	CTR
*S. aureus n* = 9	S	3 (33.3)	6 (66.6)	7 (77.7)	4 (44.4)	5 (55.5)	1 (11.1)	1 (11.1)	7 (77.7)	5 (55.5)
	I	1 (11.1)	1 (11.1)	0 (0.00)	1 (11.1)	1 (11.1)	0 (0.00)	0 (0.00)	0 (0.00)	2 (22.2)
	R	5 (55.5)	2 (22.2)	2 (22.2)	4 (44.4)	3 (33.3)	8 (88.8)	8 (88.8)	2 (22.2)	2 (22.2)

*P. aeruginosa n* = 6	S	5 (83.3)	3 (50)	4 (66.7)	ND	1 (16.6)	ND	0 (0.0)	2 (33.3)	1 (16.6)
	I	0 (0.0)	0 (0.00)	0 (0.00)		0 (0.0)		0 (0.0)	0 (0.00)	1 (16.7)
	R	1 (16.7)	3 (50)	2 (33.3)		5 (83.3)		5 (100)	4 (66.7)	4 (66.7)

*Klebsiella spp*. *n* = 5	S	3 (60)	4 (80)	3 (60)	ND	ND	ND	ND	2 (40)	4 (80)
	I	0 (0.00)	0 (0.00)	1 (20)					1 (20)	0 (20)
	R	2 (40)	1 (20)	1 (20)					2 (40)	1 (20)

*E. coli n* = 4	S	2 (50)	3 (75)		1 (25)	ND	ND	1 (25)	1 (25)	1 (25)
	I	1 (25)	0 (0.00)	ND	0 (0.00)			1 (25)	1 (25)	0 (0.00)
	R	1 (25)	1 (25)		3 (75)			2 (50)	2 (50)	3 (75)

S- Sensitive, I- Intermediate, R- Resistant, GEN- gentamicin, CIP- ciprofloxacin, AMA- amikacin, DOX- doxacillin, PEN- pencillin, FEP- cefepime, AMP- ampicillin, CTR-ceftriaxone, CAF- chloromphenical, ND- not determined.

**Table 4:** Antimicrobial resistance pattern of surface-borne bacterial isolates over ICU and OT at WSUCH, Wolaita Sodo, Ethiopia, 2021.

Antimicrobials and their effect on bacterial isolates										
Bacterial isolate	Pattern	GEN	CIP	AMA	DOX	CEP	CAF	PEN	AMP	CTR
*S. aureus n* = 38	S	12 (32.4)	23 (62.2)	18 (46.6)	18 (48.6)	20 (54.1)	26 (70.3)	4 (10.8)	35 (94.6)	25 (67.6)
	I	2 (5.4)	2 (5.4)	4 (10.8)	0 (0.00)	7 (18.9)	1 (2.7)	0 (0.0)	0 (5.4)	1 (2.7)
	R	23 (62.2)	12 (32.4)	15 (40.6)	19 (51.4)	10 (27.0)	10 (27.0)	33 (89.2)	2 (2.6)	11 (29.7)

*P. aeruginosa* [28]	S	9 (33.3)	25 (89.2)	10 (35.7)	ND	5 (17.85)	4 (14.3)	ND	18 (64.9)	15 (53.6)
	I	2 (7.1)	1 (3.6)	2 (7.1)		3 (10.7)	1 (3.6)		3 (10.7)	3 (10.7)
	R	17 (60.7)	3 (10.7)	16 (57.1)		20 (71.4)	23 (82.1)		7 (25)	10 (35.7)

*K. pneumoniae* [19]	S	13 (68.4)	16 (84.2)	12 (63.1)	ND	13 (68.4)	ND	ND	ND	11 (57.9)
	I	1 (5.3)	1 (5.3)	2 (10.5)		1 (5.3)				2 (10.5)
	R	5 (26.3)	2 (10.5)	5 (26.3)		5 (26.3)				6 (31.6)

*E. coli* [10]	S	6 (60)	7 (70)	2 (20)	2 (20)	7 (70)	ND	6 (60)	8 (80)	ND
	I	1 (10)	0 (0.00)	1 (10)	0 (0.0)	1 (10)		1 (10)	1 (10)	
	R	3 (30)	3 (30)	7 (70)	8 (80)	2 (20)		3 (30)	1 (10)	

S- Sensitive, I- Intermediate, R- Resistant, GEN- gentamicin, CIP- ciprofloxacin, AMA- amikacin, DOX- doxacillin, PEN-penicillin, CEP- cefepime, AMP- ampicillin, CTR- ceftriaxone, CAF- chloramphenicol, ND- not determined.

## Data Availability

All relevant data are within the article, but any additional data required are available from the corresponding author upon request.

## Abbreviations

ICU: Intensive care unit
MDR: multidrug resistant
OT: operation theater
TMP-SXT: trimethoprim-sulfamethoxazole
WSUCH: Wolaita Sodo University Comprehensive Hospital.
